# Myeloid-Derived Suppressor Cells (MDSC) in the Umbilical Cord Blood: Biological Significance and Possible Therapeutic Applications

**DOI:** 10.3390/jcm11030727

**Published:** 2022-01-29

**Authors:** Nikoleta Bizymi, Anthie Georgopoulou, Natalia Mastrogamvraki, Angelos Matheakakis, Ioanna Gontika, Irene Fragiadaki, Irene Mavroudi, Helen A. Papadaki

**Affiliations:** 1 Department of Haematology, University Hospital of Heraklion, 71500 Heraklion, Crete, Greece; nikoletabizymi@yahoo.gr (N.B.); a.matheakakis@gmail.com (A.M.); eirimav@gmail.com (I.M.); 2 Haemopoiesis Research Laboratory, School of Medicine, University of Crete, 71003 Heraklion, Crete, Greece; 3 Public Cord Blood Bank of Crete, University Hospital of Heraklion, 71500 Heraklion, Crete, Greece; anthieg87@yahoo.com (A.G.); nataliamast96@gmail.com (N.M.); giannagont@gmail.com (I.G.); fragiada@med.uoc.gr (I.F.)

**Keywords:** myeloid-derived suppressor cell (MDSC), umbilical cord blood (UCB), feto-maternal immune-tolerance, immunology, infection, autoimmunity, inflammation, immunotherapy, graft-versus-host disease (GVHD)

## Abstract

Myeloid-derived suppressor cells (MDSCs) represent a heterogeneous population of myeloid cells that suppress immune responses in cancer, infection, and trauma. They mainly act by inhibiting T-cells, natural-killer cells, and dendritic cells, and also by inducing T-regulatory cells, and modulating macrophages. Although they are mostly associated with adverse prognosis of the underlying disease entity, they may display positive effects in specific situations, such as in allogeneic hematopoietic stem cell transplantation (HSCT), where they suppress graft-versus-host disease (GVHD). They also contribute to the feto-maternal tolerance, and in the fetus growth process, whereas several pregnancy complications have been associated with their defects. Human umbilical cord blood (UCB) is a source rich in MDSCs and their myeloid progenitor cells. Recently, a number of studies have investigated the generation, isolation, and expansion of UCB-MDSCs for potential clinical application associated with their immunosuppressive properties, such as GVHD, and autoimmune and inflammatory diseases. Given that a significant proportion of UCB units in cord blood banks are not suitable for clinical use in HSCT, they might be used as a significant source of MDSCs for research and clinical purposes. The current review summarizes the roles of MDSCs in the UCB, as well as their promising applications.

## 1. Introduction: Myeloid-Derived Suppressor Cells

Myeloid-derived suppressor cells (MDSCs) are a heterogeneous population of myeloid cells with the ability to suppress various types of immune responses [1]. The immunosuppressive properties of myeloid progenitor cell types in the bone marrow (BM) have long been described, since 1987, in a lung cancer model by Young et al. [2]. However, the phenotypic characteristics and biological roles of MDSCs were clarified only recently. This population of cells is mainly characterized by their myeloid origin, their immature state, and their ability to suppress T-cell response [3].

In mice, MDSCs are defined by the expression of the surface markers CD11b and Gr-1 (Ly6G/Ly6C), whereas Gr-1 antigen is not expressed in humans [4]. MDSCs in humans are characterized by the CD11b^+^CD33^+^HLA-DR^−/low^ phenotype, and consist of two main subpopulations, namely the granulocytic or polymorphonuclear MDSCs (Gr- or PMN-MDSCs), and the monocytic MDSCs (M-MDSCs); an early MDSC (eMDSC) population is considered the precursor of both subtypes. The PMN-MDSCs appear similar in phenotype to neutrophils, and express the granulocytic markers CD15 and CD66b, the M-MDSCs express the monocytic surface antigen CD14, whereas the eMDSCs express neither CD15 nor CD14 [5]. It should be mentioned that there is not an absolute phenotypic distinction between PMN-MDSCs and neutrophils. However, PMN-MDSCs are low-density cells with characteristic suppressive functions. Recently, the lectin-like oxidized low-density lipoprotein receptor-1 (LOX-1) has been reported as a novel marker that further differentiates PMN-MDSCs from other low-density cells [6]. A detailed analysis of the identification of MDSCs in mice and humans is presented in this Special Issue by Vanhaver et al. [7].

Under normal conditions, the immature myeloid cells generated in the BM are rapidly differentiated into granulocytes, macrophages, or dendritic cells (DCs) [3]. However, under abnormal conditions, the immature myeloid cells expand as a result of a partial blockage of their differentiation. This is the first step required for the generation and accumulation of MDSCs. The responsible factors for this step include transforming growth factor beta (TGF-β); interleukin (IL)-1, IL-6, IL-10, IL-12, IL-13; granulocyte macrophage colony stimulating factor (GM-CSF); prostaglandin E2 (PGE2); macrophage colony stimulating factor (M-CSF); cyclooxygenase-2 (COX2); and vascular endothelial growth factor (VEGF) [8,9,10]. The second step requires a proper microenvironment for the activation of MDSCs, and acquisition of their suppressive character through the influence of certain factors, such as interferon-γ (IFN-γ); TGF-β, IL-1β, IL-4, IL-6, IL-10, IL-13; tumor necrosis factor-α (TNF-α); COX-2; hypoxia including factor-1α (HIF-1α); and Toll-like receptor (TLR) ligands [8,11]. 

The accumulation of MDSCs was initially described under conditions of malignancy where MDSCs inhibit different cell types, such as T-cells, Natural Killer (NK) cells, DCs, and macrophages [12,13,14]. In different types of tumors, cancer cells produce factors involved in the myelopoiesis (VEGF, GM-CSF, IL-1β, IL-6, HIF-1α, TGF-β, COX-2), as well as in the recruitment (CCL2, CCL3) and activation (TNF-α, IL-10, IL-1β, IL-6, INF-γ, COX-2, HIF-1α) of MDSCs [8,11]. In addition, MDSCs promote the proliferation and differentiation of Foxp3^+^ T regulatory cells (Tregs) and tumor-associated macrophages (TAM), which act in favor of tumor progression [15]. 

Studies in mice demonstrated that MDSCs suppress CD8^+^ T-cells through cell-to-cell contact, and CD4^+^ T-cells via non-specific humoral immune mechanisms [16,17]. The most common of the different suppressive mechanisms of MDSCs is the depletion of essential nutrients (L-arginine, L-cysteine, tryptophan) for T-cell proliferation via the expression of arginase-1 (Arg-1), inducible nitric oxide synthase (iNOS), and indoleamine-2,3-dioxygenase (IDO) [18]. Other mechanisms include the upregulation of COX-2, and the production of peroxynitrite (PNT), which cause nitration of the T-cell receptor-CD8 complex [19,20]. Except from their immunosuppressive functions, MDSCs play an emerging role in angiogenesis, thus contributing to tumor proliferation and metastasis via the secretion of matrix metalloproteinase (MMP-9), TGF-β, VEGF, and basic fibroblast growth factor (bFGF) [12]. 

In contrast to previous reports characterizing MDSCs as deleterious cells, there is current evidence suggesting that these cells can display either a positive or negative immune-regulatory role depending on the microenvironment and the situation. Thus, Pastula et al. characterized them as “double-edged sword” [21], whereas Budhwar et al. described them as having both “Yang and Yin” functionality [22]. Surprisingly, even in cancer, MDSCs may have anti-tumor activity through a direct neoplasmatic cells phagocytosing capacity, via interactions with T- and NK cells, and production of cytokines and effector molecules, such as NO, which, in small concentrations, has anti-tumor rather than pro-tumor effects [21]. 

Accumulation of MDSCs with opposing effects has also been described in conditions other than cancer, namely infections, trauma, obesity, autoimmune diseases, ageing, and transplantation [23]. MDSCs are beneficial during trauma and stress, as they prevent tissue damage through attenuating the excessive activation of the immune system [21]. Probably the most profound example where this function is needed is transplantation, where MDSCs prevent the rejection of the allograft [24]. Elevated levels of MDSCs have been described in the adipose tissue, and may contribute to the increased risk of cancer development in obese individuals; however, their presence may also have a beneficial role by inhibiting the metabolic deregulation observed in obesity [25]. Several studies have indicated that the accumulation of MDSCs in infections from *Staphylococcus aureus* and *Mycobacterium tuberculosis*, and in systemic lupus erythematosus, is associated with poor prognosis. In contrast, the abundance of MDSCs has been associated with good prognosis and protection from *Pseudomonas aeruginosa* and *Klebsiella pneumonia* infections, and from auto-immune conditions, such as rheumatoid arthritis and immune-related colitis [22,26]. In accordance with the aforementioned contradictory effects, pregnancy is another condition where MDSCs show not only their “bad”, but also their “good” side, as will be extensively presented below [22]. 

## 2. Immune Cells and MDSCs in Pregnancy, Fetal-Maternal Cross-Talk, and Neonatal Period

Pregnancy is a natural situation in which the mother’s immune system develops tolerating mechanisms to prevent rejection of the “allograft” fetal tissue. The maternal immune system provides an immune milieu at the feto-maternal interface, allowing the expression of paternal antigens by the fetus [26,27,28,29]. Dynamic changes between trophoblast and decidual immune cells are needed for the successful implantation and the development of the fetus [11]. Several studies demonstrate that, in pregnant women, CD4^+^ T-cells are biased toward Th2-responses, and CD8^+^ T-cells are short-lived compared to non-pregnant women [30]. Dysfunction of the immune adaptation during pregnancy usually leads to many abnormal conditions, such as pregnancy loss, preterm birth, pre-eclampsia, and fetal growth restriction [26]. 

The feto-maternal cross-talk occurs in the placenta, which is composed of both fetal and maternal cells. The Human Leucocyte Antigen (HLA)-G molecule, which is expressed by extravillous trophoblasts (EVTs) of the fetus, plays an important role in implantation and immune tolerance by modulating the immune responses of NK cells, T-cells, DCs, macrophages, innate lymphoid cells, as well as MDSCs [31]. Moreover, sex hormones, and the balance between anti-inflammatory (TGF-β, IL-10) and pro-inflammatory (IL-17) cytokines, also play distinct roles in the maintenance of pregnancy [31,32]. The cytokine balance is adjusted dynamically during the different phases of pregnancy. More specifically, in the first and third trimester, inflammatory responses are essential for the implantation and the successful fetus delivery, respectively. In contrast, regulatory responses are the major immunological events in the second trimester [33].

The number of studies investigating the role of MDSCs in pregnancy is growing recently. MDSCs participate in pregnancy as important immune-regulatory cells, although it is unclear how these cells migrate to the feto-maternal interface. Mauti et al. were the first who described that the accumulation of MDSCs in pregnancy increase the tumor metastasis in mice [13]. Several studies demonstrate increased frequency of MDSCs in all stages of pregnancy in both human and animal models. Köstlin-Gille et al. have shown that the number of PMN-MDSCs were increased ten-fold in the peripheral blood (PB) of pregnant women compared to non-pregnant, whereas the numbers of M-MDSCs were unchanged. The higher numbers of PMN-MDSCs were observed during early gestation, and dropped to the levels of non-pregnant women a few days after labor [34]. Moreover, Nair et al. have described that the numbers of MDSCs in the PB and endometrium were 30% decreased in women that experienced miscarriage compared to healthy pregnant women [35]. 

The main role of PMN-MDSCs in feto-maternal tolerance is to produce immunosuppressive enzymes (Arg1, IDO, iNOS), and to suppress T-cells through ROS production. PMN-MDSCs from the placenta exhibit increased levels of ROS compared to those from the PB [34]. On the other hand, M-MDSCs, although expressing STAT1, Arg1, and iNOS, showed less production of ROS [28]. Several studies in mice have shown that, in the absence of MDSCs, the proliferation of DCs and T-cells is increased, whereas NK cells and macrophages alone are not capable of supporting a successful pregnancy [11]. 

Besides the suppression of T-cells, MDSCs modulate the polarization of Th-cells. Many studies have shown predominant Th2 and simultaneously suppressed Th1 responses during pregnancy. More specifically, PMN-MDSCs are capable of promoting Th2 responses, and inhibiting Th1 responses in a cell-to-cell-contact-dependent manner [36]. In addition, it is well documented that MDSCs induce Tregs via the production of TGF-β and the transcription regulator β-catenin [37]. Surprisingly, Ren et al. observed an increase in the number of Tregs when depleting MDSCs in an allogeneic-normal-pregnant mouse model. NK-cytotoxicity was also altered by MDSCs via the inhibition of perforin-cytotoxicity, and the decrease of the surface receptor NKG2D on NK cells [38]. 

In terms of hormones, it is known that progesterone is essential for pregnancy maintenance by preparing the endometrium for embryo implantation [39]. Progesterone and signaling via the signal transducer and activator of transcription-3 (STAT3) increase the number and the suppressive functions of PMN-MDSCs. The activation of the progesterone receptor leads to higher expression of STAT3 in MDSCs, which in turn results in the expansion of MDSCs during pregnancy [40]. However, only one study has demonstrated a positive correlation between M-MDSCs and estrogen and progesterone during pregnancy through the STAT3 signaling pathway [41]. Moreover, other factors, such as HLA-G and HIF-α, are also responsible for the expansion and differentiation of MDSCs via the STAT3 signaling pathway in a similar manner to the aforementioned hormones [11].

One million newborns die each year worldwide due to infections [42,43]. The vulnerability of neonates and young children to infections is related to the immune system changes during this period of life [44,45,46]. It is thought that alterations in different cell types, such as monocytes, DCs, and NK cells, are responsible for this vulnerability to infections [47]. The main characteristics of this period include reduced CD8^+^ T-cell responses, accumulation of Tregs, and immaturity of DCs [48,49]. In neonates, CD4^+^ T-cells are biased towards Th2 responses, and CD8^+^ T-cells are short-lived [49], whereas several studies have shown that increased numbers of MDSCs play a key role as immuno-regulatory cells in fetuses and neonates [50]. It has been shown that PMN-MDSCs are increased in the neonates, especially during infection, presenting antimicrobial properties [51]. Specifically, neonatal PMN-MDSCs are capable of phagocytosing bacterial pathogens, but also maintain their immunοsuppressive properties [52,53]. Overall, there is a rather controversial literature on whether MDSCs are protective for fetal infections or not. Using mice and human specimens, He et al. also proved the antibacterial potential of MDSCs in infants, and showed that lower numbers of MDSCs were correlated with higher risk of death from severe infection [54]. On the other hand, Rieber et al. and Köstlin et al. argued that the increased number of PMN-MDSCs in neonates may, in part, be the reason of the impaired response to infection during this period of life [50,55]. Ongoing research on the topic will clarify the role of MDSCs in the neonatal period.

## 3. Immune Cells and MDSCs in the Umbilical Cord Blood (UCB)

The umbilical cord is the link between the fetus and the placenta. It adheres to the fetal part of the placenta; it has a mean length of 60 cm at term; and contains three vessels, i.e., two arteries and one vein, surrounded by the Wharton’s jelly [56]. Wharton’s jelly is a tissue composed from connective matrix and mesenchymal stem cells (MSCs) that protects the vessels, and ensures the unhindered transfer of elements from and to the fetus [57]. The UCB has gained particular interest as an alternative source of hemopoietic stem cells (HSCs) for transplantation (HSCT) in hematologic diseases. The HSC collection is simple and non-invasive, and the procedure is less frequently associated with graft-versus-host disease (GVHD) compared to BM-based HSCT, and thus, the HLA-matching requirements may be less strict [58]. Since the first UCB HSCT in 1988 to a patient with Fanconi’s anemia, a significant number of UCB banks have been established to provide candidate transplants all over the world [59,60]. 

Most of the HSCs present in the fetal circulation, i.e., in the placenta and the umbilical cord, migrate to the BM after delivery; although, some may remain in the above tissues. Interestingly, the concentration of HSCs in the UCB is higher compared to the BM or PB [58]. Furthermore, the UCB stem and progenitor cells have longer telomeres and different gene expression profiles, and they can easily expand ex vivo even without growth factors, as they have the ability to produce them in an autocrine manner [60,61]. Τhe immature CD34^+^rh123^low^CD38^−^ cells, and those expressing Thy-1, present the highest proliferation rates in cultures [60]. In contrast, MSCs are present in low numbers in UCB, whereas the identified endothelial cell populations probably derive from the umbilical cord rather than the UCB [58]. 

The reduced risk of GVHD in HSCT of UCB-derived HSCs compared to BM is probably associated with the unique characteristics of the UCB immune cells to achieve the feto-maternal tolerance [62]. Although DCs are present in UCB, most of them do not express CD11c, and they are not capable of stimulating T-cells and initiating the immune responses, though they are capable of inducing CD4^+^CD25^+^ Tregs. Compared to the PB, the UCB DCs express lower levels of MHC class II, CD80, and CD86 [62]. The T-cells of the UCB are mostly naïve, with an absence of the cytotoxic and memory phenotype; they express low levels of CD40 ligand and perforin; and present higher apoptosis rates [60,61]. Similarly, NK cells express low levels of CD57, and present reduced cytotoxicity [60,62]. Tregs, on the other hand, are abundant in the UCB, and fully functional, and thus, infusion of UCB Tregs is a promising novel treatment of GVHD [62]. 

The numbers of MDSCs in the UCB and the PB of neonates are increased compared to adults, and decrease during the first months of life [50]. The number of UCB MDSCs are comparable to those observed in cancer patients [43]. It is unclear whether this accumulation of MDSCs in neonates is beneficial or may cause susceptibility to infections. Many studies have demonstrated that the higher amount of MDSCs observed in the umbilical cord of preterm neonates may contribute to the higher risk to infections [51]. However, other studies suggest that MDSCs may protect neonates from uncontrolled inflammatory responses, as they produce higher levels of anti-microbial agents [52,54]. 

**Figure 1:**
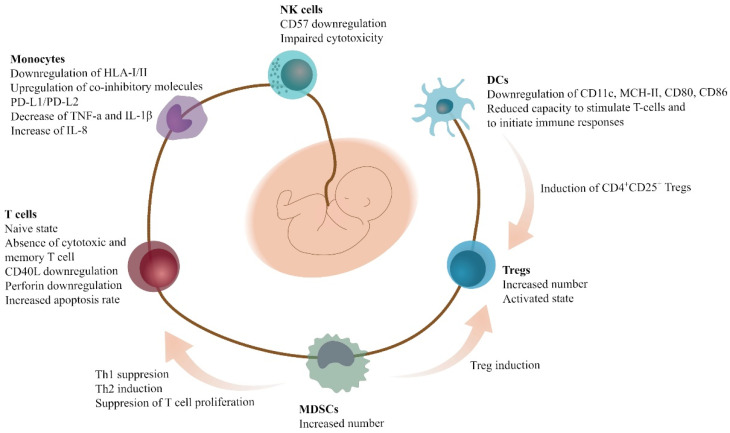
Specific characteristics and interactions of different immune cells in the UCB. Immune cells found in the UCB exhibit distinct features, leading to complex immune regulation patterns. Inhibition of immune activation is elicited in multiple manners that implicate MDSCs, among others, finally shifting the balance towards a tolerant microenvironment. Abbreviations: UCB, umbilical cord blood; CD40L, CD40 ligand; DCs, dendritic cells; HLA, human leukocyte antigen; IL, interleukin; MDSCs, myeloid-derived suppressor cells; NK cells, natural-killer cells; PD-L; programmed death ligand; Th: T-helper cells; Tregs, T-regulatory cells; TNF, tumor necrosis factor.

The majority of MDSCs in neonates are PMN-MDSCs, and are capable of suppressing the proliferation of T-cells. UCB MDSCs are capable of suppressing Th1 responses, and inducing Th2 responses and Tregs. Th1 responses are mediated through cell-to-cell contact, whereas Th2 responses are mediated through the production of Arg1 and ROS. The induction of Tregs is mediated through iNOS expression [26]. Other studies have shown that UCB PMN-MDSCs have effects on monocytes. More specifically, PMN-MDSCs are responsible of the downregulation of HLA class I and class II molecules; the upregulation of co-inhibitory molecules, such as programmed death ligand-1 (PD-L1) and PD-L2; the decrease of TNF-a and IL-1β; and the increase of IL-8 [63]. The interactions and properties of the UCB immune cells are depicted in Figure 1. 

### 3.1. In Vitro and Ex Vivo Expansion of UCB-MDSCs

Given their low numbers, the in vitro and ex vivo expansion of MDSCs for potential therapeutic purposes, such as GVHD and prevention of autoimmunity, remains a challenge [1,64,65]. The UCB contains high numbers of precursor cells, and can thus serve as an excellent source for this purpose. In the following paragraphs and in Table 1, the main studies involving the expansion of UCB-MDSCs and their results are summarized.

Yu et al. induced the generation of MDSCs by co-culturing UCB-CD33^+^ cells with MDA-MB-231 breast cancer cells [66]. The CD45^+^CD33^+^CD13^+^CD14^-^CD15^-^ cells were defined as induced MDSCs (iMDSCs). The iMDSCs possessed the same immunosuppressive properties as fresh MDSCs from cancer patients. The generated cells induced apoptosis of T-cells, decreased the T-cell secretion of IFN-γ, and increased the T-cell secretion of IL-10 and TGF-β. The authors showed that the phosphorylation of STAT3 and the following up-regulation of IDO are essential for the immune-modulatory actions of iMDSCs, by blocking these molecules/pathways, as well as by comparing iMDSCs with untreated UCB-CD33^+^ cells. The use of iMDSCs in this study highlights the contact-dependent manner of the immunosuppression of MDSCs, and points MDSC-associated targets (molecules and pathways) for novel therapies [66]. 

Wu et al. cultured UCB-CD34^+^ cells in HSC medium containing GM-CSF, granulocyte-colony stimulating factor (G-CSF), and/or IL-6 [67]. This combination of factors was essential for the generation of CD11b^+^CD14^+^HLA-DR^−/low^ cells. Moreover, these factors were important for the up-regulation of important immunosuppressive molecules in the precursor cells facilitating the differentiation to MDSCs, such as M-CSF receptor (M-CSFR), IL-4 receptor-α (IL-4Rα), PD-L1, Arg-1, and CCAAT/enhancer-binding protein-β (C/EBPβ). The UCB-MDSCs that were treated with these factors suppressed the proliferation of CD3^+^, CD3^+^CD4^+^, or CD3^+^CD4^−^ T-cells, and the production of IFN-γ from T-cells, and altered the expression of PD-L1 and CD3e from T-cells and forkhead box P3 (FoxP3), the marker of Tregs. With this study, the authors gave evidence that the aforementioned cytokines, also produced from solid tumors, lead to the transition of the HSC to an immature myeloid cell, and, subsequently, by acquiring the suppressive character, to MDSCs [67].

Park et al. also generated MDSCs from UCB-CD34^+^ cells cultured with recombinant human (rh) GM-CSF/stem cell factor (SCF), G-CSF/SCF or M-CSF/SCF [68]. The most effective combination was the rh-GM-CSF/SCF. After 6 weeks of culture with rh-GM-CSF/SCF, the cells expressed the markers specific for M-MDSCs, i.e., HLA-DR^low^, CD11b^+^, CD33^+^, CD14^+^, CD15^-^, as well as the typical signaling and suppressive molecules, i.e., STATs, mammalian target of rapamycin (mTOR), protein kinase B (PKB, Akt), Arg-1, IDO, iNOS, IL-10, and VEGF. According to the authors, the generated cells suppressed the proliferation of CD4^+^ and CD8^+^ T-cells, and led to the induction of FoxP3^+^ Tregs. Moreover, the generated cells reduced the secretion of IFN-γ in the co-cultures of DCs and CD4^+^ T-cells. The authors tested the generated cells in a mouse acute GVHD (aGVHD) model, where the infused MDSCs displayed a protective role, and improved the survival of mice through the aforementioned mechanisms in vivo, suggesting that these cells could be used in the treatment of human inflammatory diseases [68]. 

Lim et al. cultured human UCB-CD34^+^ cells with rh-GM-CSF and rh-SCF [69]. The generated cells showed increased numbers of CD14^+^HLA-DR^low^CD11b^+^CD33^+^ populations, and elevated Arg-1 and iNOS, but not NADPH oxidase 2 (NOX2) expression. The authors adoptively transferred these cells to two mouse models of chronic GVHD (cGVHD). MDSCs could be detected in vivo in the skin, lung, and spleen. The generated MDSCs suppressed the proliferation of both mouse and human CD4^+^ and CD8^+^ T-cells in vitro. When treated with MDSCs, the mice suffered less from cGVHD, and the damage in the organs was decreased, including the thymus, which presented less expansion of donor-type T-cells. Attenuated was also the damage and expansion of T-cells following G-CSF treatment in all tissues, including the spleen, skin, and lung, among others. MDSCs changed the balance in the cytokine production in the recipients by down-regulating the production of IL-17 and IL-4 from CD4^+^ T-cells, and by up-regulating the production of IFN-γ from CD4^+^ T-cells, and increasing FoxP3^+^ Tregs, favoring Th1 over Th2/Th17 differentiation. The findings of this study show that MDSCs generated ex vivo from human UCB could be used in future treatment regimens not only against aGVHD, but also against cGVHD [69]. 

Zoso et al. [70] and Mazza et al. [71] generated a novel population of MDSCs, named fibrocytic MDSCs (f-MDSCs), which co-expresses markers of MDSC, tolerogenic DCs (tDCs), and fibrocytes, i.e., CD33, IL-4Rα, CD11b, CD11c, CD13, CD14, CD15, HLA-DR, CD86, CD40, collagen V, and a-smooth muscle actin (a-SMA). Moreover, the transcriptional analysis of the cells depicted that they were a unique population relative to MDSCs, but neither M- nor PMN-MDSCs, sharing markers and having a profile between the three types of cells, i.e., MDSCs, tDCs, and fibrocytes. The cells were obtained by a 4-day culture of UCB with rh-GM-CSF and rh-G-CSF. The generated cells had a fibrocyte-like morphology and adhesive properties. The authors proved the suppressive character of f-MDSCs, which is mediated via the induction of Tregs by IDO up-regulation upon contact of f-MDSCs with T-cells rather than by the expression of Arg-1 or NOS2. This novel population delayed the onset of Type 1 Diabetes when adoptively transferred in a mouse model. The authors propose this novel subset as a tool for the treatment of allograft rejection and in vitro generation of Tregs [70,71].

### 3.2. Applications of UCB-MDSCs: Experimental and Clinical

MDSCs have drawn the attention of scientists for the development of novel therapeutic strategies involving blockage of their development, differentiation, depletion, deactivation, or their adoptive transfer, as well as the recognition of novel MDSC-associated biomarkers for personalized treatment approaches [1,64,65]. 

MDSCs displaying T-cell and NK cell suppressive function, and contributing to feto-maternal tolerance, are increased in the UCB and in the fetus, and decrease gradually in childhood. The increased MDSC cell population in UCB is that of PMN-MDSCs [31,43,53,72,73]. The PMN-MDSCs in the UCB are different from the placenta; they are heterogeneous in phenotype, and are affected by several external factors [74]. Thus, the UCB-MDSCs and molecules implicated in their immunosuppressive functions, such as Arg-1, have been proposed as biomarkers of failure of feto-maternal tolerance [31,43,51,72,73]. Moreover, the fact that MDSCs, and specifically PMN-MDSCs, fail to increase in the appropriate levels in preeclampsia, intrauterine growth restriction, spontaneous abortion, and preterm birth, suggests that they could not only serve as possible predictive biomarkers, but also as therapeutic targets [11,28,75].

The increase in MDSCs has been associated with the ineffective response to infections in the postnatal period. Interestingly, during infections, the levels of PMN-MDSCs are further elevated, and are correlated with biochemical markers of inflammation [51]. In preterms, the increased MDSC numbers may have a beneficial role in the blockade of uncontrolled inflammatory responses; however, it may also predispose to infections [11,51]. Leiber et al. showed that PMN-MDSCs play a double role upon infection with *Escherichia coli*, i.e., potent phagocytes that can eliminate pathogens as first line defense and anti-inflammatory regulators [52]. Dietz el al. co-cultured UCB-MDSCs and monocytes, and described that MDSCs modulated the monocyte-associated markers and production of TNF-α, but also inhibited their phagocytosis capacity [63]. Heinemann et al. showed the association of S100A8/S100A9 alarmins, important molecules for MDSC function, with septic shock and fatal sepsis in neonates by using human UCB samples and a septic mouse model [76]. Accordingly, the PMN-MDSCs and the associated molecules can act as biomarkers and therapeutic targets in severe infections and sepsis to improve the defense during the neonatal period [50,55].

The immunosuppressive function of MDSCs is important to tackle the host inflammatory responses responsible for GVHD and autoimmunity [66,67,68,69,70,71]. The fact that MDSCs suppress T-cell responses, and promote the expansion of Tregs, is beneficial against GVHD [77]. As described above, experimental data have shown that ex-vivo-expanded UCB-MDSCs display beneficial effects in animal models of aGVHD, cGVHD, and type 1 diabetes associated with autoimmune responses [66,67,68,69,70,71]. 

Furthermore, novel applications are currently under consideration. McDonald et al. treated mice that suffered hypoxic-ischemic brain injury by intraperitoneally infusing UCB cells. UCB cells were shown to have a neuroprotective effect by reducing inflammation and cell death [78]. The study suggests the possible use of UCB cells in the management of perinatal brain injury. Although these results can be partially mediated via MDSCs, further research on this field is needed [78]. 

**Figure 2:**
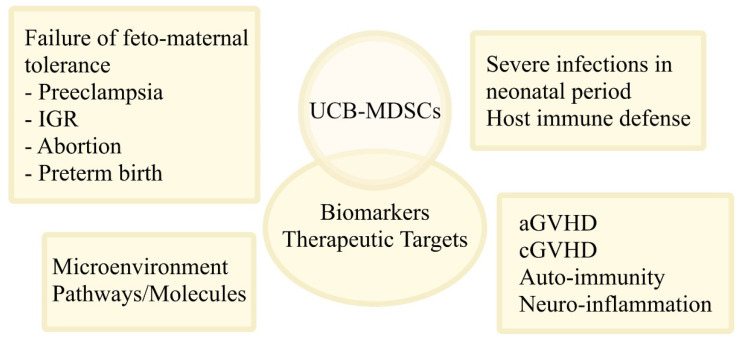
The role and potential applications of UCB-MDSCs. The use of UCB-MDSCs in clinical practice is currently under investigation. The MDSCs and molecules related to their function may be used as biomarkers or/and therapeutic targets in failure of feto-maternal tolerance (i.e., preeclampsia, IGR, abortion, preterm birth), in severe infections in neonatal period to facilitate the host immune defense in this period, in aGVHD and cGVHD, in auto-immunity, and in neuro-inflammation. UCB-MDSCs are also involved in immune interactions within different microenvironments, including the umbilical cord, implicating cell-to-cell contacts and a variety of pathways and molecules. Abbreviations: UCB, umbilical cord blood; MDSCs, myeloid-derived suppressor cells; IGR, intra-uterine growth restriction; aGVHD, acute graft-versus-host disease; cGVHD, chronic GVHD.

The interaction of MDSCs with umbilical cord cells is an ongoing field of research. Yang et al. showed that co-cultures of peripheral blood mononuclear cells (PBMCs) and UCB-MSCs result in the MSC-mediated proliferation of MDSCs via the HLA-G [79]. Qi et al. showed that co-cultures of mice MDSCs with umbilical cord MSCs result in the MSC-induced immaturity of PMN-MDSCs via PGE2, and of M-MDSCs via IFN-β [80]. Morton et al. simulated the human tumor microenvironment by generating mismatched humanized mice via infusion of human UCB HSC/progenitor cells and UCB-MSCs [81]. After the implantation of head and neck squamous cell carcinoma (HNSCC) tumors, an expansion of mice MDSCs was identified that concerned mainly the early stage MDSC populations [81]. A summary on the potential applications of UCB-MDSCs is presented in Figure 2. 

## 4. Unpublished Data from the Public Cord Blood Bank of Crete

In an attempt to characterize the UCB-MDSCs for potential future therapeutic applications, we are evaluating the number of MDSCs in UCB of full-term pregnancies, as well as factors that may affect the frequency of their populations, in the Public Cord Blood Bank of Crete of the University Hospital of Heraklion, Crete, Greece. The MDSC subpopulations have been so far measured in the mononuclear cell fraction of 40 UCB units from full-term pregnancies (37 to 41 weeks) and the respective maternal PB samples using flow cytometry as previously described [1]. Our preliminary, unpublished data show that the proportion of UCB PMN-MDSCs and M-MDSCs correlate with the frequency of the respective maternal cell populations, the proportion of UCB-CD34^+^ cells, the neonatal weight, the UCB unit volume, and the week of gestation, whereas it does not depend on the sex of the newborn. In our next steps, we will study the functional characteristics of UCB-MDSCs. It is well known that a significant proportion of UCB units are not suitable for clinical use in HSCT [82]. Maternal, neonatal, and obstetric factors have an impact on the quality of the UCB collections, and the most common reasons for inappropriateness are delivery complications, compromised collection bag integrity, incomplete or inappropriate labeling, positive infectious disease testing, unacceptable quality control indexes, and low total nucleated cell count and/or low volume [83]. Given that a number of these UCB units may be used for research purposes [84], including their potential use as a source for MDSC isolation, the knowledge of the parameters that affect the number and the qualitative characteristics of UCB-derived MDSCs are of particular importance.

## 5. Conclusions

MDSC populations, in contrary to what was believed in the past, play an immune-modulatory role that can be either positive or negative depending on the microenvironment and the situation in which these cells grow. They display a beneficial effect during pregnancy, as increased numbers of MDSCs are crucial in the development of immunological tolerance of the fetus. UCB is a source rich in MDSCs, and UCB-MDSCs are gaining particular interest for potential clinical uses, such as, among others, in allogeneic HSCT for GVHD inhibition. As UCB-MDSCs are candidate cells for several promising applications because of their immunomodulatory potential, the UCB units that are not suitable for clinical use in HSCT represent an attractive source for MDSC isolation for research and clinical purposes. 

## Figures and Tables

**Table 1:** Studies involving in vitro and ex vivo expansion of UCB-MDSCs.

Study	Progenitor Cells-Origin	Technique	Generated Cells	Importance
Yu et al. [66]	UCB-CD33^+^ cells-human	Co-culture with MDA-MB-231 human breast cancer cells	CD45^+^CD33^+^CD13^+^CD14^−^CD15^−^ cells	Identification of (a) the contact-dependent manner of the immunosuppression of MDSCs and (b) targets, i.e., MDSCs, molecules, and pathways for possible novel therapies
Wu et al. [67]	UCB-CD34^+^ cells-human	Culture with GM-CSF with G-CSF and/or IL-6	CD11b^+^CD14^+^HLA-DR^-/low^ cells	Cytokines produced from solid tumors lead to the transition of the hematopoietic stem cells to immature myeloid cells, and, subsequently, to MDSCs with suppressive character
Park et al. [68]	UCB-CD34^+^ cells-human	Culture with rh-GM-CSF/SCF	HLA-DR^low^CD11b^+^CD33^+^CD14^+^CD15^-^ cells	Evidence that generated MDSCs can be used in the treatment of aGVHD and human inflammatory diseases
Lim et al. [69]	UCB-CD34^+^ cells-human	Culture with rh-GM-CSF and rh-SCF	CD14^+^HLA-DR^low^CD11b^+^CD33^+^ cells	MDSCs generated ex vivo from human UCB can be used in treatment regimens not only against aGVHD, but also against cGVHD
Zoso et al. [70], Mazza et al. [71]	UCB cells-human	Culture with rh-GM-CSF and rh-G-CSF	f-MDSCs (co-express markers of MDSC, tDCs, and fibrocytes, i.e., CD33, IL-4Rα, CD11b, CD11c, CD13, CD14, CD15, HLA-DR, CD86, CD40, collagen V, and a-SMA)	f-MDSCs can serve as a tool for treatment of allograft rejection and in vitro generation of T regulatory cells

Abbreviations: UCB—umbilical cord blood; MDSCs—myeloid-derived suppressor cells; CD—cluster of differentiation; HLA-DR—human leukocyte antigen-DR isotype; GM-CSF—granulocyte-macrophage-colony-stimulating factor; G-CSF—granulocyte-CSF; IL—interleukin; rh—recombinant human; SCF—stem cell factor; f-MDSCs—fibrocytic-MDSCs; tDCs—tolerogenic dendritic cells; IL-4Ra—IL-4 receptor-a; a-SMA—a-smooth muscle actin; aGVHD—acute graft-versus-host disease; cGVHD—chronic GVHD.

## Data Availability

The data presented in this study are available on request from the corresponding author. The data are not publicly available as the study is ongoing.
